# Co-modulation of a circular form of PCDH11Y during neuroendocrine differentiation of prostate cancer

**DOI:** 10.3389/fonc.2025.1502405

**Published:** 2025-02-11

**Authors:** Giovanni Pecoraro, Ilaria Leone, Silvia Nuzzo, Santiago Negueruela, Giovanni Smaldone, Lorena Buono

**Affiliations:** ^1^ IRCCS SYNLAB SDN, Naples, Italy; ^2^ Telethon Institute of Genetics and Medicine (TIGEM), Pozzuoli, Italy

**Keywords:** circRNA, prostate cancer, biomarker, neuroendocrine, trans-differentiation

## Abstract

**Introduction:**

Prostate cancer (PC) is a leading cause of cancer-related deaths among men, often progressing to castration-resistant prostate cancer (CRPC) after androgen deprivation therapy (ADT). A subset of CRPC evolves into treatment-emergent neuroendocrine prostate cancer (t-NEPC), an aggressive form characterized by poor prognosis. Currently, there is no reliable biomarker for early detection of t-NEPC. Circular RNAs (circRNAs) have emerged as potential biomarkers due to their stability and tissue-specific expression.

**Methods:**

In this study, we investigated the circRNA landscape during neuroendocrine transdifferentiation (NED) of PC cells using the androgen-sensitive LNCaP and androgen-insensitive DU145 cell lines. To achieve that, we applied CirComPara2 pipeline to publicly available datasets to identify the differently expressed circRNAs in the LNCaP cell lines pre- and post-transdifferentiation. After that, validation and functional analysis by RNA-interference was applied to a selected circRNA to explore its role during NED.

**Results:**

We identified over 6,200 circRNAs, of which 33 were differentially expressed during NED. Among them, a novel circRNA, circPCDH11Y, was highly upregulated during the transition of LNCaP cells from an epithelial to neuroendocrine phenotype, while its levels remained unchanged in DU145 cells. Functional assays demonstrated that circPCDH11Y plays a role in regulating the expression of key neuroendocrine markers, including synaptophysin (SYP), neuron-specific enolase (ENO2), prostate-specific antigen (PSA), Brain-Specific Homeobox/POU Domain Protein 2 (BRN2) and the linear form of Protocadherin 11 Y-Linked (PCDH11Y). Silencing circPCDH11Y delayed the expression of SYP, ENO2 and PCDH11Y, while increasing PSA and BRN2 transcriptional levels, indicating its involvement in promoting neuroendocrine differentiation. Additionally, circPCDH11Y was detected in extracellular vesicles (EVs) secreted by LNCaP cells post-NED, suggesting its potential as a circulating biomarker.

**Discussion:**

These findings highlight circPCDH11Y as a promising candidate for early detection of t-NEPC and provide new insights into the molecular mechanisms underlying prostate cancer progression. Further validation in clinical samples is required to establish its diagnostic and therapeutic potential, which could significantly improve the management of treatment-resistant prostate cancer.

## Introduction

Prostate cancer (PC) accounts for more than 200,000 new cases each year and is considered the most common cause of neoplasm in man other than the second leading cancer-related death cause in the developed world (1). PC is a strictly age-related disease, with the risk increasing over the 55 years of age (2). In addition to this, it has been observed that prostate cancer does not show an even distribution across ethnicities, with African-Americans more affected by the onset of the disease and more susceptible to its lethality (3). Genetics also plays a key role in the onset of the disease, with men with close relatives suffering from PC having about twice the probability to develop this neoplasm (4). PC diagnosis classification is based on the combination of several indicators, including circulating PSA levels, Gleason Score (GS) and tumour node metastasis (TNM), as well as the ‘*in situ*’, ‘advanced’ or ‘metastatic’ nature of the tumour (5). Although localized prostate cancer has a slow growth rate and can be eradicated by simple surgical resection or remain under active surveillance, in the case of advanced stage diagnosis, treatment options include combining surgery coupled to androgen deprivation treatment (ADT) and/or chemotherapy (6). Initially, prostate carcinomas depend on androgenic activity to develop, which is why they are sensitive to ADT even in the early-metastatic phase. ADT therapy is based on the administration of LHRH agonist or antagonists, so to prevent secretion of LH and in turn reduce testicular production of androgens (7). This therapy could eventually be supplemented by the blockage of adrenal residual androgens, resulting in a “combined androgen blockage” treatment (8, 9).

Despite ADT therapy remaining the treatment of choice for PC patients, drug-resistance often emerges causing castration-resistant disease (CRPC), a tumoral phenotype whose regulatory mechanisms could be mediated by aberrant activity of androgen receptor (AR) signaling pathway (10–13). Alternatively, the process of resistance to therapies that target the androgen receptor and cause CRPC may be mediated by altered androgen sensitivity, causing cells to become AR-independent and evolve towards a more aggressive phenotype, known as treatment-emergent neuroendocrine prostate cancer (t-NEPC) (14–16). Neuroendocrine cells (AR/PSA negative) are often present in small number within luminal prostate tumors, and their ability to grow without androgen stimulation cause them to be enriched within the tumor after ADT therapy cycles (17, 18). In most cases, NEPC emerges as a transdifferentiation process of prostate cancer cells during ADT treatment (t-NEPC), with some rare exceptions (0.5-2% of primary tumors) in which the disease directly emerges from pre-existing neuroendocrine cells within the prostatic gland, causing an extremely aggressive variant of the neoplasm (19). Moreover, there is a subset of CRPC recurrent cases (17-30%) which show an histology entirely composed by neuroendocrine cells (small cell neuroendocrine carcinoma, or SCNC) which carries the poorest prognosis among all prostate cancer subtypes (20, 21).

From a diagnostic point of view, a histopathologic classification of neuroendocrine differentiation and a definition of NEPC has been proposed, in which the major diagnostic criteria of t-NEPC is the finding of small or large cell neuroendocrine carcinoma in patients that underwent ADT (22, 23). Immunohistochemistry (IHC) markers of NEPC often include positivity for insulinoma-associated protein 1, CD56, synaptophysin, CgA and neuron-specific enolase (ENO2) (16, 24, 25). Clinical features of t-NEPC include rapid progression, low PSA/tumor burden ratio, and poor prognosis due to unresponsiveness to therapies (22). Moreover, t-NEPC shows higher serum levels of LDH and NSE compared to metastatic castration-resistant carcinoma (mCRPC), albeit these markers demonstrated low specificity and positive predictive value (26). From a transcriptional regulation perspective, Brain-Specific Homeobox/POU Domain Protein 2 (BRN2) and Achaete-Scute Family BHLH Transcription Factor 1 (ASCL1) have been identified as master regulator of neuroendocrine differentiation process in PCa (27, 28).

Currently, there’s no standard of care for t-NEPC. Most therapeutic attempts are based on chemotherapy with cytotoxic agents, the use of immune checkpoint inhibitors (albeit with poor results) or targeted agents, as in the case of NEPC with AURKA overexpression treated with the inhibitor alisertib (29).

All considered, clearly identifying t-NEPC by using clinical criteria is still extremely challenging, causing clinical suspicion which in turn claims for repeated biopsies in patients with clinically aggressive disease, increasing the urge for the seek of validated, reliable biomarkers. In the oncological field, circular RNAs (circRNAs) are emerging as promising biomarkers due to their stability and tissue-specific expression. They have been found to be dysregulated in many diseases, including cancer (30), offering potential for non-invasive diagnostics and targeted therapies, enhancing personalized medicine approaches (31). circRNAs are single-stranded RNAs, generally produced during mRNA splicing process, that form a covalently closed continuous loop because the 3′ and 5′ ends are joined together creating a back-splicing site; for this reason, they are resistant to exonuclease activity and more stable than other non-coding RNAs (ncRNAs) (32). Between the many functions ascribed to circRNAs there are miRNA sponging, competing with mRNA splicing, and modulating the transcription or post-transcription of target genes (33). As for many other type of neoplasms, circRNAs are also gaining increasing importance for their multifaced roles in prostate cancer (34–36).

In this work, we characterized the landscape of circular RNAs in AR-dependent (epithelial phenotype) and AR-independent (neuroendocrine phenotype) transdifferentiated form of LNCaP PC cell line. Additionally, we discovered a novel circular RNA not previously associated with PC, originating from a back-splicing event of the PCDH11Y gene. Our analyses revealed that circPCDH11Y is highly upregulated during the process of transdifferentiation from epithelial prostate cancer to neuroendocrine prostate cancer in different cell line models. Moreover, we explored the presence of circPCDH11Y in LNCaP-secreted EVs before and after the transdifferentiation process, in order to assess its possible use as circulating biomarkers. Finally, we functionally characterized circPCDH11Y by silencing its expression and analyzing its regulatory effects on the other neuroendocrine (NE) markers during the transdifferentiation. Overall, our results propose a new, consistent biomarker that could help the early diagnosis of t-NEPC and, in turn, improve effective clinical interventions.

## Materials and methods

### Cell lines

Androgen-dependent LNCaP (37) metastatic prostate carcinoma cell line and androgen-independent prostate carcinoma DU145 cell line (38) were purchased from ATCC (American Type Culture Collection) and grown in phenol red-containing Roswell Park Memorial Institute (RPMI) 1640 Medium (Gibco – Fisher Scientific) supplemented with 10% FBS (GE Healthcare), 1× Pen-Strep (Lonza) and 2 mm l-Glutamine (Lonza). Cells were kept in a humidified incubator at 37°C in the presence of 5% CO2 and all experiments were performed under conditions of exponential growth. Cells were STR authenticated and periodically tested for the presence of mycoplasma contamination using MycoBlue Mycoplasma Detector D101 (cat: D101-01; Vazyme Biotech).

To induce neuroendocrine differentiation, LNCaP cells were allowed to grow for 2 to 3 passages prior to harvesting and plated to a final confluence of 50%. After 24 hours, medium was discarded, cells were washed twice with PBS and a steroid-depleted medium composed of RPMI 1640 w/o phenol red (Gibco – Fisher Scientific) supplemented with 10% charcoal-stripped FBS (cat: A3382101, Gibco – Ficher Scientific), 1× Pen-Strep (Lonza) and 2 mM l-Glutamine (Lonza) was added. To induce neuroendocrine differentiation in DU145 cells, complete medium was replaced by RPMI supplemented with 2% FBS, 2 mM L-Glutamine and 50 ng/mL of Epidermal Growth Factor (EGF) (cat: AF-100-15; Preprotech, USA) and refreshed daily until day 7, as described by Humez et al. (39).

### Omics characterization of the circular RNA landscape

Deep sequencing of rRNA-depleted total RNAs of AR-dependent and AR-independent LNCaP growth state was retrieved from GSE114052 (40). CircRNA landscape was characterized as in Altieri et al. (41). Briefly, circRNA isoforms were detected *de novo*, annotated and quantified using CirComPara2 (42), a computational pipeline to detect, quantify, and correlate expression of linear and circular RNAs from RNA-seq data that combines multiple circRNA-detection methods. GRCh38 was used as reference genome for the analysis. The complete list of detected circRNAs can be found in **Supplementary Dataset S1**. Differential circRNA expression was calculated with DESeq2 starting from Circompara2 resulting circRNA raw counts, using a p-value less than 0.05 as significance threshold (43). The complete list of detected circRNAs can be found in **Supplementary Dataset S2**. Gene ontology enrichment was assessed using enrichR (44), using the complete list of significantly differentially regulated circRNAs of **Supplementary Dataset S2**. Complete output of the ontology enrichment analysis have been added as **Supplementary Datasets S3**, **S4**.

### RNA extraction and quantitative PCR

At the timepoint of interest, cells were detached, harvested and centrifuged at 3000 rpm for 5 minutes at 4°C. After washing the pellet once with cold PBS, cells were resuspended in 1 mL of Qiazol (cat: 79306; Qiagen) and lysed for 5 minutes at room temperature (RT). For the evaluation of circRNA in EVs, EVs pellet resulted after ultracentrifugation was resuspended in Qiazol and 0.5 pmol/ml of CL4 aptamer (5′-GCCUUAGUAACGUGCUUUGAUGUCGAUUCGACAGGAGGC-3′) was added as reference control. In both procedures, subsequently, 200 uL of chloroform were added and the tubes were vortexed and incubated at RT for 10 minutes. Following centrifugation at 12.000 g for 15 minutes at 4°C, the aqueous phase was collected in a new tube and an equal volume of isopropanol was added; the mixture was subsequently incubated for 10’ at 4°C and centrifuged at 12.000 g for 30minutes at 4°C. Finally, supernatants were discarded, and pellets were washed once with ethanol 70%, dried and resuspended in DEPC-treated water for further analysis. RNA purity and quantification was assessed using the Implen™ NanoPhotometer™ NP80 Nano-Volume and Cuvette UV-VIS Spectrophotometer (Implen).

RNA was retrotranscribed using the SuperScript™ IV VILO™ Master Mix kit (cat: 11756050; Invitrogen – Fisher Scientific) according to manufacturer instructions. Generated cDNA was then processed for qPCR using the iQ SYBR^®^ Green Supermix (cat: 1708882; Biorad) and gene expression was evaluated by normalizing the Ct values of target genes on RPS18 reference gene (for whole cell RNA extracts) or by comparing circPCDH11Y Ct versus CL4 Ct (for EVs RNA extracts), prior proceeding to confronting between the different treatments.

Primers used to evaluate gene expression list as follow:

TUBB3 For 5’-GAT CGG GGC CAA GTT CTG T-3’
TUBB3 Rev 5’-GCC TCG TTG TAG TAG ACG CT-3’
PSA For 5’-CGT GAC GTG GAT TGG TGC T-3’
PSA Rev 5’-ACC CAG CAA GAT CAC GCT TT-3’
SYP For 5’-TGG GGA CTA CTC CTC GTC AG-3’
SYP Rev 5’-GTG GCC AGA AAG TCC AGC AT-3’
ENO2 For 5’-TGC ACA GGC CAG ATC AAG AC-3’
ENO2 Rev 5’-CCA GGC AAG CAG AGG AAT CA-3’
CL4 For 5′-GCCTTAGTAACGTGCTTT-3′
CL4 Rev 5′-GCCTCCTGTCGAATCG-3′
ASCL1 For 5’- CAA GCA AGT CAA GCG ACA GC-3’
ASCL1 Rev 5’- TTG ACC AAC TTG ACG CGG TT-3’
BRN2 For 5’- GTT GCC GTT TTG GGG GAT TT-3’
BRN2 Rev 5’- ACG AAG AAG GGG CAA CAC AA-3’
PCDH11Y For 5’-CAA CTC CGA TCC TGA ATC TAC TTT-3’
PCDH11Y Rev 5’-CTT CCA CAG TTG GTT GAA CAG T-3’
circPCDH11Y_For 5’-CGA TAA CAC CTT TGT GGC CTG-3’
circPCDH11Y_Rev 5’-TTT TAA GCA CCC TCG GTC TGG T-3’

### circPCDH11Y backsplicing site sequencing

To obtain the amplicons of circPCDH11Y backsplicing sites, RNA from 18 days neuroendocrine transdifferentiated cells was extracted and retrotranscribed using the method above described (see Materials and Methods, RNA extraction and quantitative PCR section). Subsequently, cDNA was selectively amplified using target circRNA primer couple and Platinum SuperFi II DNA Polymerase (cat: 12361010; Invitrogen-Fisher Scientific), according to manifacturer instructions. After that, the amplification product was loaded on a 2% agarose electrophoresis gel and run until a single band of the correct bp length was visible. The band was excised from the gel and nucleic acids were purified using QIAquick PCR Purification Kit (cat: 28104; Qiagen). Assessment of DNA fragments concentration and purity was performed using the Implen™ NanoPhotometer™ NP80 Nano-Volume and Cuvette UV-VIS Spectrophotometer (Implen). Sample was then sent to Eurofins Genomics (Louisville, Kentucky, USA) for Sanger sequencing (Sequencing Order: 11108159108-1) and results were sent back as FASTA format.

### Cell transfection and functional evaluation of circPCDH11Y

LNCaP cells were seeded at a density of 500,000 cells/well 24 hours prior transfection in a 6-well plate, so to reach a final density of 60%. On the day of transfection, cells were treated with 10 nM of three circPCDH11Y DsiRNA mix (Sales Order 3969702) or, alternatively, with 10 nM of negative control DsiRNA, using RNAiMAX (Thermo Fisher Scientific, USA) following manufacturer instructions. All DsiRNAs were designed and purchased from IDT (Coralville, Iowa; USA) giving predicted circRNA sequence to the technical support.

24hrs after transfection, treated cells were gently washed twice with PBS and hormone-depleted medium was added to initiate the neurodifferentiation process. Medium was refreshed every 2 days, and cells were collected and lysed at the selected timepoints [24hrs post transfection (0), after 4 days of neurodifferentiation (4) and after seven days of neurodifferentiation (7)] using Qiazol to proceed to the evaluation of the expression of the selected markers (see Materials and Methods, RNA extraction and quantitative PCR section).

Each experiment was performed in biological triplicates.

### Cell viability assay

24 hours after being transfected with circPCDH11Y DsiRNA mix or negative control DsiRNA, cells were harvested and an aliquot of 50 µL of cell suspension was taken from each biological replicate to be further processed. Briefly, 5 µL of propidium iodide (PI) (cat:6607055, Beckman Coulter, USA) was added to the suspension and incubated in the dark for 5 minutes at RT. After that, 1 mL of PBS was added to each tube to dilute PI, cells were next pelleted and resuspended in 200 µL of PBS, prior being analysed by cytofluorimetry using the Cytoflex flow cytometer (Beckman Coulter, USA). A suspension of complete medium-grown LNCaP cells was used to set the threshold to ECD positivity. 10,000 events were taken into consideration for each replicate. Data were subsequently analyzed using Kaluza software (Beckman Coulter, USA) and the percentage of PI positive cells were compared among the different groups of treatment. Each experiment was performed in biological triplicates.

### Cell cycle assay

24 hours after being transfected with circPCDH11Y DsiRNA mix or negative control DsiRNA, cells were harvested and pelleted. Briefly, each pellet was fixed and stained for PI using the Coulter DNA Prep Reagents kit (cat:6607055, Beckman Coulter, USA). Fixation lasted 1 hour at RT, while PI staining was carried out in the dark for 3 hours at RT. Subsequently, the samples analysed by cytofluorimetry using the Cytoflex flow cytometer (Beckman Coulter, USA). 20,000 events were taken into consideration for each replicate. Data were subsequently analyzed using Kaluza software (Beckman Coulter, USA) and differences of cell cycle phase distributions among the different groups were examined. Each experiment was performed in biological triplicates.

### EVs isolation and EVs circPCDH11Y and linearPCDH11Y amplicon analysis

EVs were isolated from approximately one million of LNCaP cells and LNCaP neurodifferentiated at 4- 7 and 21 days.

Ultracentrifugation was performed to isolate EVs from conditioned medium after 72 hours, as previously described in MISEV2023 guidelines (45).

Specifically, medium from all cell lines was centrifuged at 300 x g for 10 minutes to remove cells, then the supernatant was centrifuged at 2000 x g for 10 minutes to remove dead cells. Larger EVs were removed by ultracentrifugation at 10,000 x g for 30 minutes at +4°C. Cleared conditioned medium was then ultracentrifuged at 200,000 x g for 1 hour at +4°C for pelleting EVs. Finally, EVs pellets were washed once using sterile PBS and resuspended in 100 µL of sterile PBS for further analysis. After qPCR analysis for circPCDH11Y and linear PCDH11Y presence in the EVs (see Materials and Methods, RNA extraction and quantitative PCR section), amplification products were loaded on a 2% agarose electrophoresis gel and run until a single band of the correct bp length was visible.

### Nanoparticle tracking analysis of extracellular vesicles

Particle concentration and size of LNCaP cell derived vesicles were analyzed using NTA (NanoSight NS300, Malvern Instruments Ltd, Malvern, UK).

Samples were diluted in 200 µl filtered 1× PBS in order to obtain an optimal range of 20–150 particles/frame. Briefly, 10 μl of sample was further diluted with 1× PBS to a final volume of 1 ml (dilution factor = 1:100) and loaded into the instrument. Samples were injected into the NTA system under constant flow conditions (flow rate = 50). The instrument’s software NTA 3.4 Build 3.4.4 was used for the measurement. For each sample, 5 videos of 60″ seconds duration were recorded, and data were processed. Samples were injected into the NTA system under constant flow conditions (flow rate = 50).

### Immunoblotting analysis

Lysed EVs and cells (30 μg) were resolved on 10% Mini-PROTEAN^®^ TGX Stain-Free™ (Bio-Rad Laboratories, Cat. #4568034) at 120V and then proteins were transferred by the Trans-Blot Turbo System (Bio-Rad Laboratories, Cat. # 690BR024275). The detection of protein will do by using primary antibodies (1:1000) followed by incubation with HRP mouse or rabbit IgG (1:5000) in PBS containing 5% non-fat dry milk (Bio-Rad Laboratories). Primary antibody used: anti-TSG101 (1:1000; Cat. # ab30871) and anti-Calnexin (1:1000; Abcam) antibodies. Imaging was performed using an automated ChemiDoc™ MP Imaging System (Cat. # 12003154, Bio-Rad Laboratories) and Clarity Max™ Western ECL Substrate (Cat. # 1705062, Bio-Rad Laboratories). LNCaP whole cell lysate was used as a positive control.

## Results

### Characterization of the circRNA landscape in androgen-dependent and androgen-independent LNCaP growth state

**Figure 1:**
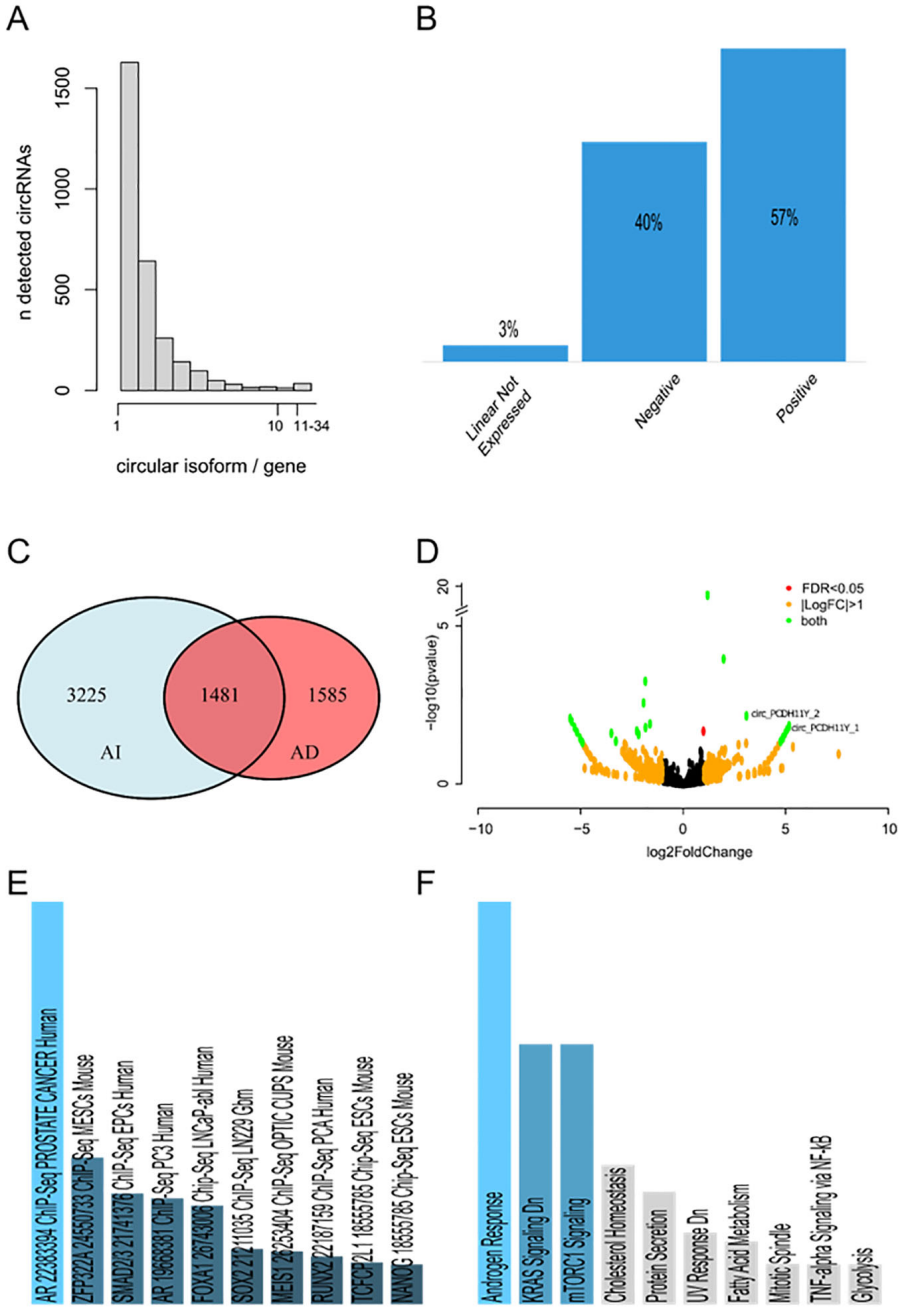
**(A)** Number of circular RNA isoforms detected for each gene. **(B)** Expression correlation of each circRNA detected with its respective linear transcript. **(C)** Venn diagram of circRNAs detected in at least one sample of AR-dependent and AR-independent LNCaP. 1585 circRNAs were exclusively detected in AR-dependent LNCaP, 3225 were exclusive to AR-independent LNCaP, while 1481 circRNAs were identified in both AR-dependent and independent LNCaP differentiation state. **(D)** Volcano plot of differentially expressed circRNAs detected by DESeq2. On the right, the circRNAs upregulated in the AI-LNCaP. On the left, the downregulated ones. CircRNAs that are differentially regulated with a log2(FoldChange) greater than 1 or less than -1, and at the same time have a false discovery rate significance less than 0.05 are highlighted in green. CircRNAs with a log2(FoldChange) greater than 1 or less than -1, but a false discovery rate significance greater than 0.05 are highlighted in orange. CircRNAs with a significant differential regulation and a log2(FoldChange) less than 1 are highlighted in red. **(E)** ChEA enrichment of differentially expressed circRNAs calculated by EnrichR. The first 10 hits ordered by significance are showed in the plot. Different shades of blue and decreased size of the bar correspond to decreasing significance. Results showed in grey are not significant (p-value greater than 0.05). **(F)** Molecular signature enriched in differentially expressed circRNAs calculated by EnrichR. The first 10 hits ordered by significance are showed in the plot. Different shades of blue and decreased size of the bar correspond to decreasing significance. Results showed in grey are not significant (p-value greater than 0.05).

We conducted a *de novo* characterization of the circRNA landscape in prostate carcinoma cell line LNCaP along neuroendocrine transdifferentiation, using two AR-dependent and two AR-independent biological replicates. Our investigation identified a total of 6291 circRNAs. Among these, 6211 circRNAs contained genomic regions associated with previously annotated linear transcripts, while 80 circRNAs could not be linked to any known linear transcript (see complete list, genomic coordinates and annotations of the circRNAs provided in **Supplementary Dataset S1** in **Supplementary Materials**). The majority of linear transcripts were linked to a single circular isoform (25.88%), and in general, most transcripts exhibited up to five circular isoforms. However, notable exceptions were observed, such as the genes TTC6 (22 circRNAs), NCAM and ASPH (23 circRNAs), UBAP2 and STXBPL5 (24 circRNAs), and LRBA (34 circRNAs), each showing more than 20 circular isoforms derived from the same linear transcript (**Figure 1A**). Among the circRNAs associated with a linear transcript, 57% exhibited a positive correlation with the expression of the linear isoform, while 40% showed an inverse correlation. In only 3% of cases, the corresponding linear transcripts were not detected as expressed (**Figure 1B**). From a qualitative perspective, 1585 circRNAs were exclusively detected in AR-dependent LNCaP, 3225 were exclusive to AR-independent LNCaP, while 1481 circRNAs were identified in both AR-dependent and independent LNCaP differentiation state (**Figure 1C**). Further, differential expression analysis identified 33 differentially expressed circRNAs, 17 upregulated in AR-dependent LNCaP, while 16 are upregulated in neuroendocrine AR-independent LNCaP (**Figure 1D**; complete list in **Supplementary Dataset S2** in **Supplementary Materials**). Enrichment results for ChEA analysis run through EnrichR (46) identified “AR 22383394 ChIP-Seq PROSTATE CANCER “ as the top-ranked term supporting the idea that input list of circRNA used is significantly enriched for binding sites of AR as a transcription factor and consistent with regulating biological processes and pathways of AR prostate cancer (**Figure 1E**). Furthermore, enrichment analysis for molecular signatures (47) revealed that the linear transcripts producing these differentially expressed circRNAs are not only associated with prostate cancer in general but are also specifically involved in the androgen response (**Figure 1F**). This specific enrichment underscores the potential regulatory role of circRNAs in mediating androgen response, contributing to the progression and treatment resistance observed in neuroendocrine androgen-independent type of prostate cancer.

### Circular PCDH11Y is overexpressed during NED in LNCaP, but not in DU145

Bioinformatic prediction identified several circular RNA which could be differentially modulated during neuroendocrine trans-differentiation (NED) of LNCaP cells. Our attention was focused on the circPCDH11Y, the circular RNA derived from PCDH11Y gene, a gene involved in the androgen-independent prostate cancer cell growth and neuroendocrine trans-differentiation (48). Initial validation of the presence of a backsplice junction in this circRNA was carried out by target PCR followed by electrophoresis gel analysis, band extraction and amplicon Sanger sequencing (**Supplementary Figure S1**). Sequencing results showed a complete sequence identity with the predicted backsplicing sites (**Supplementary Dataset S5** in **Supplementary Materials**).

**Figure 2:**
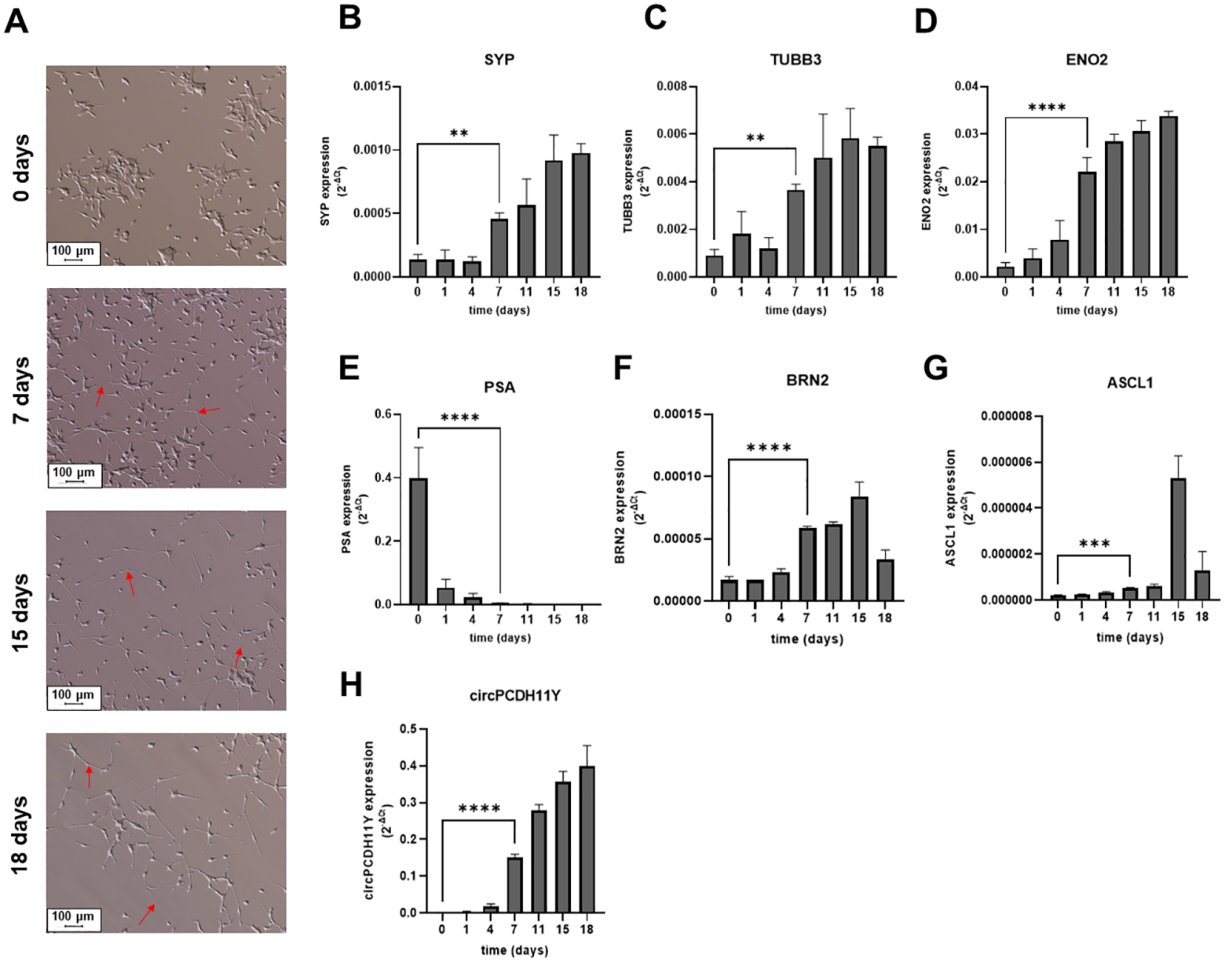
**(A)** Microscope acquisitions of LNCaP morphological changes at different stages of induced neuroendocrine transdifferentiation (10x fields), from the basal epithelial phenotype (first micrograph from the top) to the neuronal cell shape acquired during NED (second, third and fourth micrograph from the top for 7, 15 and 18 days of treatment, respectively). Red arrows indicate neuritic branches developed during transdifferentiation. Relative mRNA expression levels of validated neuroendocrine markers during different stages of LNCaP transdifferentiation **(B-G)**. Different genes were evaluated: **(B)** Synaptophysin (SYP); **(C)** Tubulin Beta 3 Class III (TUBB3); **(D)** Neuron-specific Enolase (ENO2); **(E)** Prostate specific antigen (PSA); **(F)** Brain-Specific Homeobox/POU Domain Protein 2 (BRN2); **(G)** Achaete-Scute Family BHLH Transcription Factor 1 (ASCL1). **(H)** Circular PCDH11Y was also evaluated under the same conditions. Statistical significance with respect to the starting LNCaPs is represented for all genes at day seven and calculated using an ordinary one-way ANOVA test (*p < 0.05; **p < 0.01; ***p < 0.001; ****p < 0.0001).

We induced a change in cell phenotype of LNCaP cell line from epithelial to neuroendocrine as previously reported (38). We treated LNCaP for 18 days with RPMI w/o phenol red in the absence of hormone stimulation, using 10% dextran-coated charcoal-treated FBS as medium supplement. Morphological analysis revealed a switch from the classical LNCaP epithelial phenotype (**Figure 2A**, first micrograph from the top), with the presence of many long-branched neuritic-like processes (**Figure 2A**, red arrows) which started to develop early during the trans-differentiation process and that finally resulted in a complete transformation of the cell population toward a neuronal shape already at day 7 (**Figure 2A**, second micrograph from the top), with long axons-like structures and small cell bodies that became more evident at day 18 (**Figure 2A**, third micrography from the top) and day 21 (**Figure 2A**, fourth micrography from the top). Then we verified the expression of several cellular markers closely related to the neuronal phenotype. As expected, synaptophysin (SYP), tubulin beta 3 class III (TUBB3) and neuron-specific enolase (ENO2), achaete-scute family BHLH transcription factor 1 (ASCL1) and brain-specific homeobox/POU domain protein 2 (BRN2) exhibited a gradual but constant increase over time, reaching significativity between 4 and 7 days of stimulation when compared to complete medium-grown LNCaP (**Figures 2B–D, F, G**). On the contrary KLK3 (PSA), a gene whose expression is tightly regulated by androgen receptor, underwent a drastic reduction in gene expression over the time span of the treatment (**Figure 2E**). Notably, day 7 of treatment was the time point in which every marker of neuroendocrine transdifferentiation had significant variation compared to complete medium-grown cells, indicating that timespan as sufficient to explore the early phase of NEPC transformation.

**Figure 3:**
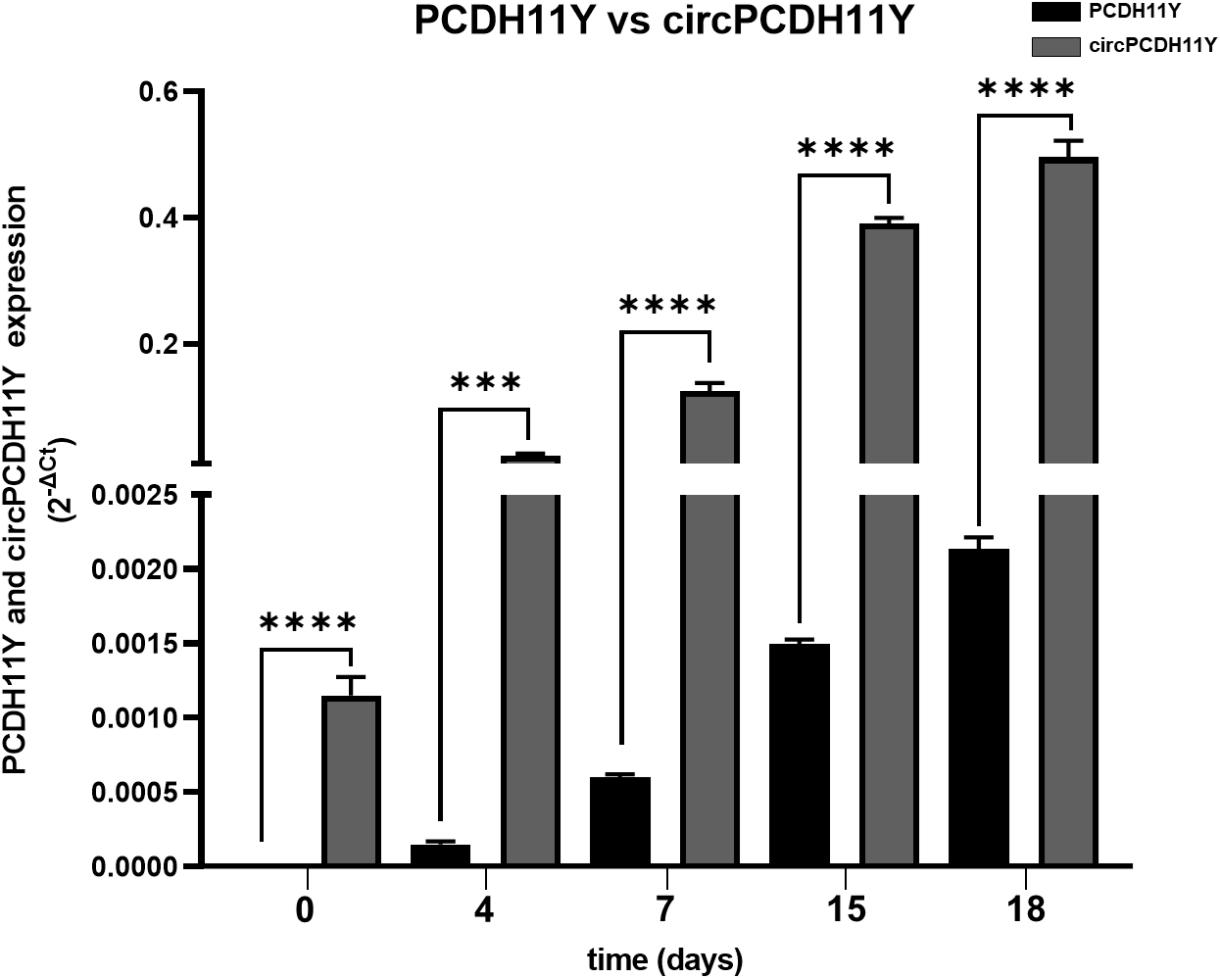
Comparison between PCDH11Y (black bars) and circular PCDH11Y (grey bars) during LNCaPs neuroendocrine trans-differentiation. Statistical significance was calculated using a parametric t-test (*p < 0.05; **p < 0.01; ***p < 0.001; ****p < 0.0001).

Surprisingly circPCDH11Y showed constant and drastic increase during neuroendocrine differentiation (**Figure 2H**), and its expression at day 7 of treatment is significantly higher respect to the day 0. Moreover, circPCDH11Y showed a fold increase of over 200 when compared to its linear mRNA form, which is an already well-established marker of prostate NED (**Figure 3**) (43).

**Figure 4:**
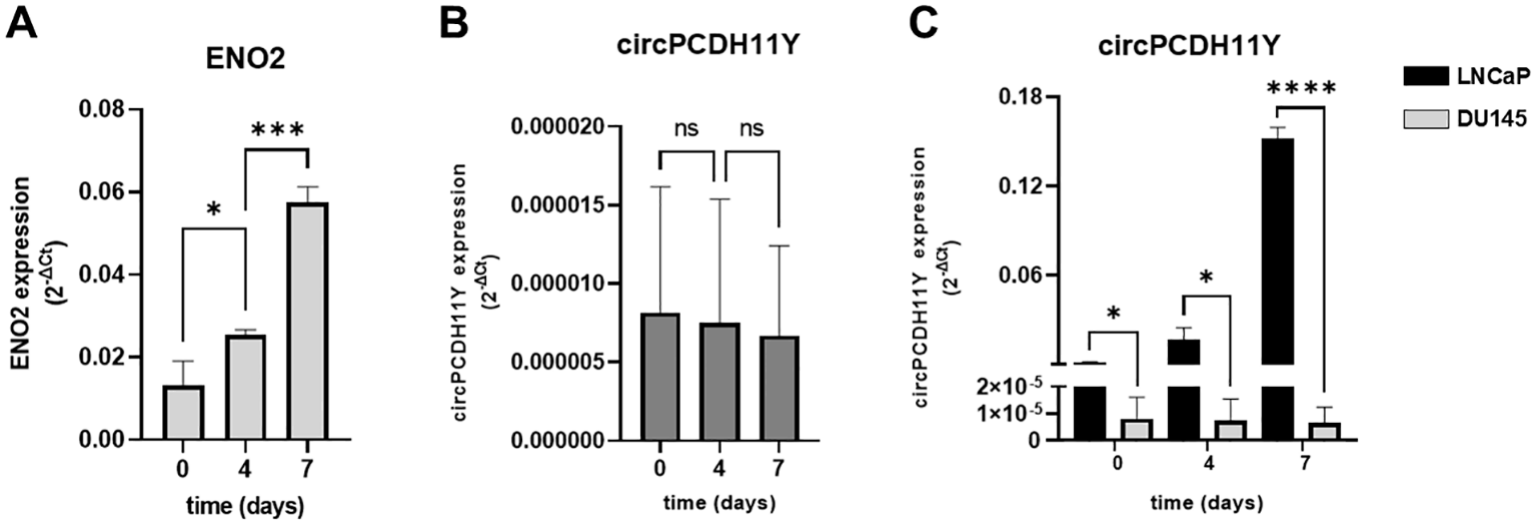
**(A)** Relative mRNA expression level of ENO2 in DU-145 cell lines after different timepoints of exposure to EGF, indicating neurodifferentiation process. **(B)** Relative mRNA expression level of circPCDH11Y in DU-145 cells during different stages of DU-145 transdifferentiation. Statistical significances are calculated using an ordinary one-way ANOVA test. **(C)** Comparison of circPCDH11Y relative mRNA levels between LNCaP and DU-145 cells at different timepoints during neurodifferentiation; statistical significance was calculated using a nonparametric Mann-Whitney test (*p < 0.05; **p < 0.01; ***p < 0.001; ****p < 0.0001; ns, statistically not significant).

We used a second NED cell model, the androgen-independent DU145 cell line, to confirm our analyses. As described by Humez et al. (39), DU145 cell line were treated at different time points (day 0,4 and 7) using 2% FBS + 50ng/mL EGF as NED inducer. Although we confirmed that the treatment caused a constant increase in ENO2 expression over the 7 days of analysis, as demonstrated by Humez and colleagues (**Figure 4A**), no alterations of the circPCDH11Y expression levels was observed over time in this case (**Figure 4B**). Furthermore, when confronting LNCaP and DU145, it appeared evident that the first model already showed higher level of circPCDH11Y at timepoint 0 (**Figure 4C**).

### circPCDH11Y expression in exosomal vescicles

**Figure 5:**
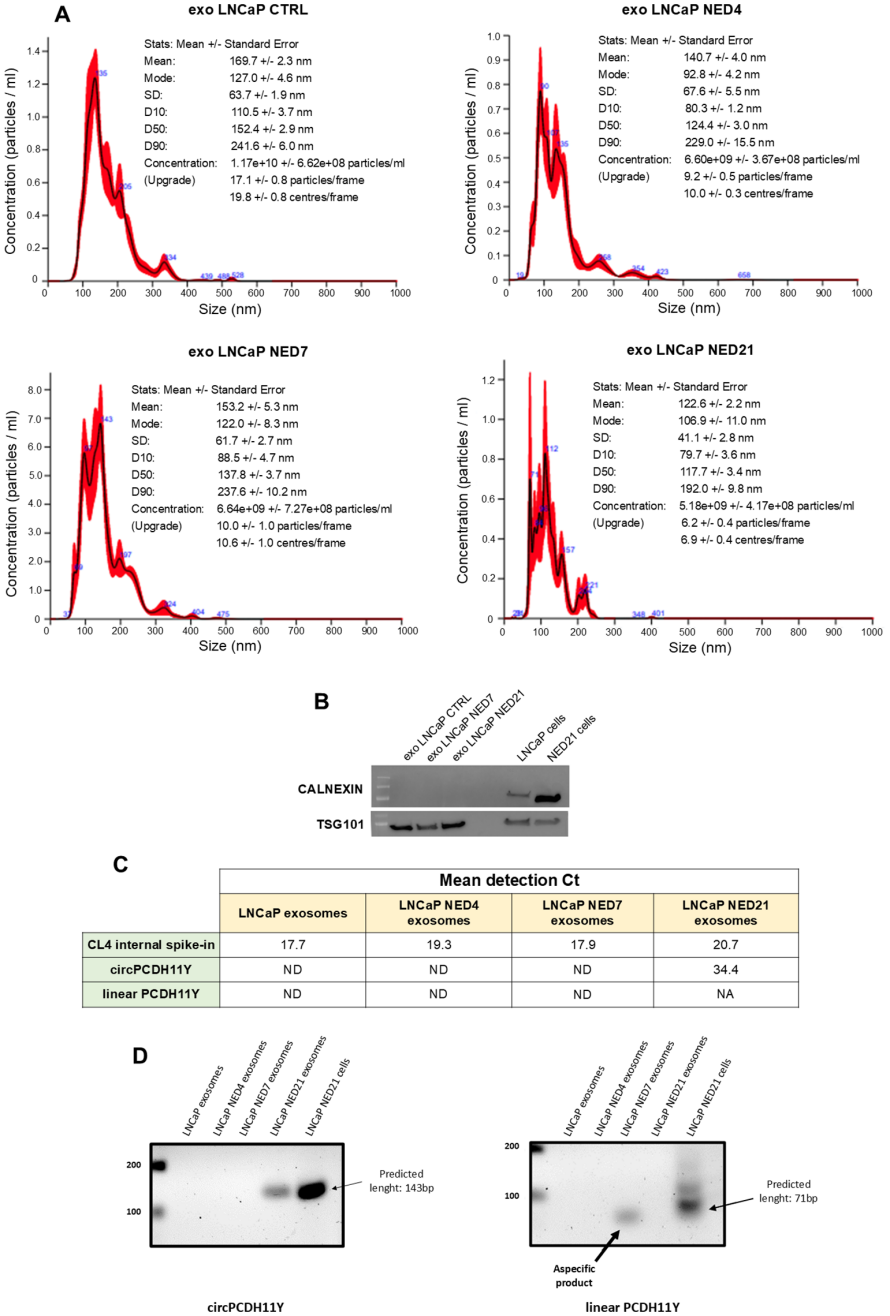
EVs isolation from LNCaP medium during different neuroendocrine-transdifferentiation timepoints and EVs circPCDH11Y presence validation at late stages. **(A)** Size distribution of isolated EVs analysed by Nanosight NS300; **(B)** Western Blot analysis of positive and negative EVs markers TSG101 and Calnexin respectively. **(C)** RT-qPCR EVs expression level of CL4 aptamer, circPCDH11Y and linear PCDH11Y at different time of NED. Expression levels are reported as Ct of RT-qPCR experiments (ND, Not Detected, refers to a signal not detected until cycle 40, endpoint of qPCR experiments). **(D)** target amplification for circPCDH11Y (right panel) and linear PCDH11Y (left panel) after EVs RNA extraction and agarose gel electrophoresis analysis. For circPCDH11Y, the amplicon of the predicted size was only visible at day 21 after NED induction. For linear PCDH11Y, an aspecific byproduct of amplification was visible at day 7, but considered ND when compared to control amplicon. Amplicons from the neurodifferentiated cell line at day 21 was carried as controls.

To date, it is clear that exosomal vescicles (EVs) contain a number of biomarkers involved in cell communication (39). LNCaP-EVs and NED-EVs were identified using Nanosight, with the main particle size ranging from 50 to 150 nm (**Figure 5A**). The WB results demonstrated that the EV surface marker TSG101 was expressed under both conditions, while the negative marker Calnexin was absent in both (**Figure 5B**, **Supplementary Figure S2**). circPCDH11Y expression in EVs was evaluated using RT-PCR. Our data demonstrate that circPCDH11Y was detected in NED-EVs (day 21) but not in LNCaP-EVs confirming the differences observed on cells (**Figure 5C**). Moreover, the presence of the linear form of PCDH11Y was also tested in the exosome, but no amplification signal was detected at NED throughout the experiment, as shown for the final timepoint (**Figure 5C**). These preliminary data indicate that circPCDH11Y may be an emerging circulating biomarker of neurodifferentiation process carried by EVs (**Figures 5C, D**).

### Silencing circPCDH11Y impacts the expression of NED markers

**Figure 6:**
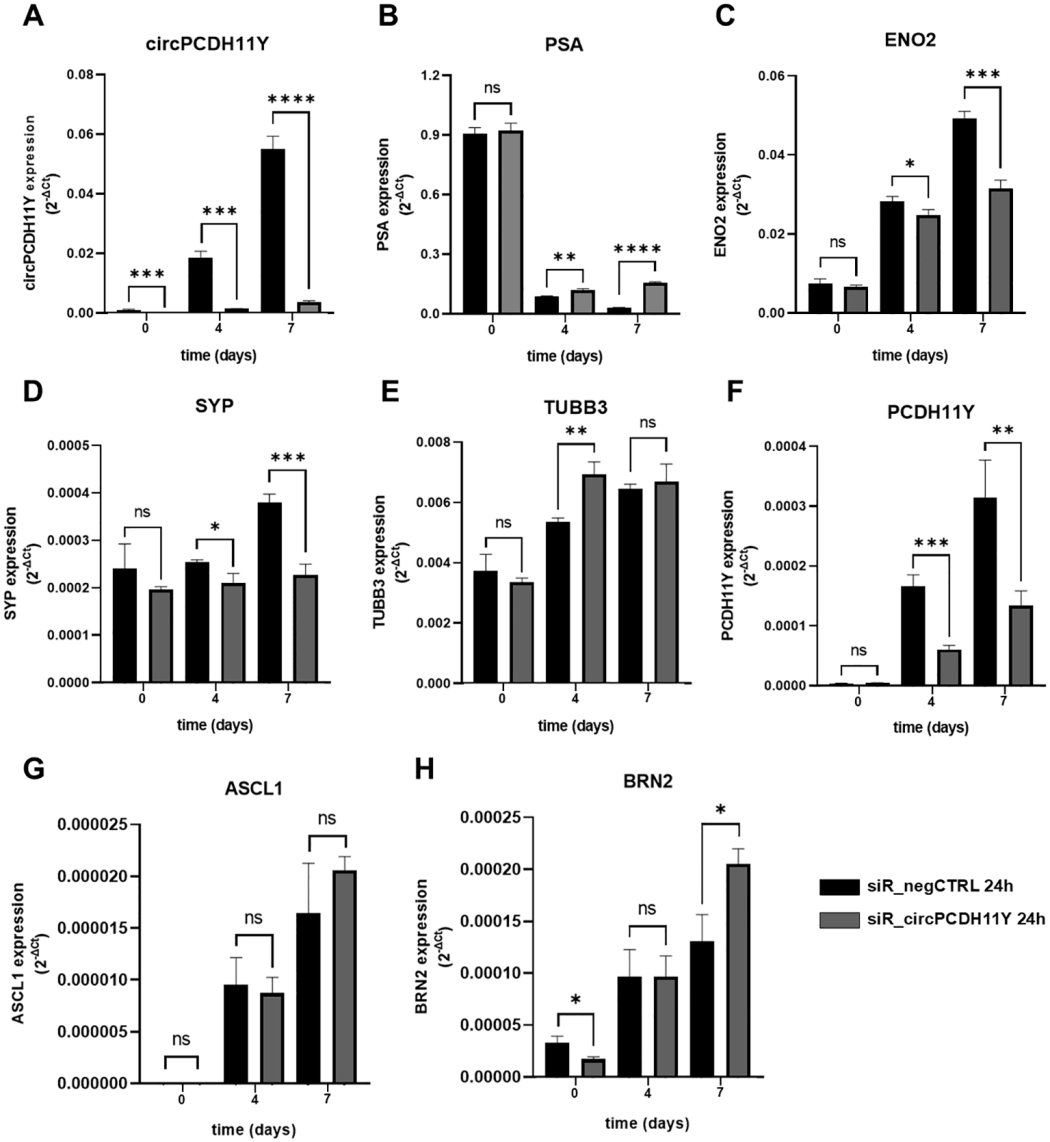
Effects of circPCDH11Y silencing on neuroendocrine differentiation of LNCaP cells. Relative mRNA expression levels were compared between siR_negCTRL treated and siR_PCDH11Y_2 treated cells at different timepoints after neurodifferentiation induction for **(A)** circPCDH11Y; **(B)** PSA; **(C)** ENO2; **(D)** SYP; **(E)** TUBB3; **(F)** PCDH11Y; **(G)** ASCL1 and **(H)** BRN2. Three independent experiments were performed to generate the data. Unpaired t-test was used to calculate the statistical significance between the two conditions at each time point (*p < 0.05; **p < 0.01; ***p < 0.001; ****p < 0.0001; ns, statistically not significant).

In order to investigate the role of circPCDH11Y in the early stages of neuroendocrine transdifferentiation process, we performed silencing experiments on the target circular RNA using an equimolar mix (10nM final) of three DsiRNAs targeting the backsplicing site of circPCDH11Y (**Supplementary Figure S3**) or, alternatively, a negative control DsiRNA on LNCaP cells for 24 hours (time point 0), prior replacing the regular culture medium with the hormone-depleted one, initiate the transdifferentiation and assess the expression of NED markers at different timepoints. The choice of targeting the backsplicing site of the identified cirRNA was aimed to exclude any possible off-target effects on the linear form of PCDH11Y. To rule out the possibility that any observed changes in target genes expression following silencing were due to alterations in cell viability or cell cycle related to DsiRNA toxicity, we first performed specific assays to analyze these two parameters (**Supplementary Figure S4**). Results indicate that the transfection did not cause any significant changes either in cell viability (**Supplementary Figure S4**, left panel) and cell cycle progression (**Supplementary Figure S4**, right panel), creating the ideal starting condition to carry on our experiments on NED regulation. Interestingly, results showed that, although a transient method has been used to negatively modulate circPCDH11Y RNA levels, the silencing remained robust and significant compared to the negative control throughout the experiment (**Figure 6A**). At time point 0, none of the known NED markers showed any significant modulation in their expression levels, except for BRN2, which resulted increased in circPCDH11Y silenced cells (**Figures 6B–G**). PSA expression drastically dropped in both treating conditions as observed at day 4 of NED, but the data indicated that while negative control treated cells had a continuous decrease of PSA mRNA levels during the timespan of the experiment, circPCDH11Y silenced cells showed an opposite trend, reaching a significantly higher expression at both day 4 and 7 (**Figure 6B**). On the other hand, we observed that ENO2 (**Figure 6C**) and SYP (**Figure 6D**) levels increased, as expected, over time in negative control treated cells, while silenced cells had a significantly lower increment at both day 4 and day 7 of NED. We observed no appreciable changes in TUBB3 and ASCL1 levels that could be linked to DsiRNA activity (**Figures 6E, G**). Finally, the silencing affect significantly reduces the expression level of the linear form of PCDH11Y, another well notes NED marker (**Figure 6F**). Interestingly, we observed a positive trend for BRN2 when cells were treated with the DsiRNA (**Figure 6H**), compared to negative control, showing that depletion of circPCDH11Y did not exert exclusively transcriptional downregulation effects. Overall, the results obtained suggest that circPCDH11Y plays an active role in the neuroendocrine transdifferentiation process, as its silencing, while not stopping NED completely, is able to modulate the expression movement of key NED markers in mostly opposite directions to what normally occurs during trans-differentiation, except for the key neuronal transcription factor BRN2.

## Discussion

For decades, circRNAs have been considered as byproducts of mRNA processing, without any particular biological significance (32). Anyway, with the advent of RNA sequencing, in silico prediction and functional analysis, thousands of circular RNAs have been subsequently found to be implicated in multiple processes as development, physiological and pathological conditions, including cancerogenesis and cancer progression. Due to their tissue-specific expression and greater stability to the action of exonucleases than canonical mRNAs, these molecules are emerging as useful biomarkers for different types of diseases, as well as therapeutic agents, because of their ability to modulate gene expression in different ways (49).

In this study, we characterized the circular RNA (circRNA) landscape during the process of neuroendocrine transdifferentiation (NED) in prostate cancer (PC) cells, particularly focusing on the androgen-dependent LNCaP cell line and the androgen-independent DU145 cell line. Our findings revealed significant differences in circRNA expression between androgen-sensitive and androgen-insensitive states, with circPCDH11Y emerging as a key player in NED. This novel circRNA, derived from the PCDH11Y gene, a gene involved a gene involved in the androgen-independent prostate cancer cell growth and neuroendocrine trans-differentiation (48, 50, 51), was highly upregulated during trans-differentiation, suggesting its potential role as a biomarker for t-NEPC (52). The identification of 6291 circRNAs, with distinct expression profiles in AR-dependent and AR-independent cells, highlights the complexity of circRNA regulation in PC progression. Importantly, 33 circRNAs were differentially expressed during neuroendocrine transdifferentiation, underscoring their potential involvement in mediating treatment resistance and phenotypic plasticity (53, 54). This is consistent with previous reports suggesting that circRNAs, through their stability and tissue-specific expression, are involved in various regulatory processes, including miRNA sponging and mRNA regulation (55, 56). Our results specifically point to the significant overexpression of circPCDH11Y during the early stages of NED in LNCaP cells, while its levels remained unchanged in DU145 cells. This data could be suggesting a correlation between the circPCDH11Y and AR status as it has been already demonstrated for its parental gene PCDH11Y (57–59), as also suggested by the ChEA enrichment analysis. Further research is needed to determine the exact role of circPCDH11Y in AR signaling during prostate cancer transdifferentiation. Moreover, functional characterization of circPCDH11Y through silencing experiments provided compelling evidence of its involvement in the regulation of neuroendocrine markers such as PSA, ENO2, and synaptophysin (SYP). Silencing circPCDH11Y resulted in delayed upregulation of ENO2 and SYP, two critical neuroendocrine markers, while PSA levels, normally reduced during NED, showed an unexpected increase; moreover BRN2, a transcription factor master regulator of neuronal differentiation, showed an inverse tendency toward upregulation when circPCDH11Y was silenced. This suggests that circPCDH11Y may play a direct role in promoting neuroendocrine differentiation by regulating downstream key markers associated with the process (60). Although NED was not completely halted by circPCDH11Y silencing, the observed delay in secondary marker expression highlights its importance in modulating the timing and progression of transdifferentiation. The ability of circPCDH11Y to modulate neuroendocrine differentiation makes it an attractive candidate for further exploration as a potential biomarker for t-NEPC. Its exclusively association with LNCaP-secreted EVs, when compared with the linear form of the parental gene, before and after NED adds to its potential utility in non-invasive diagnostics, as exosomal circRNAs could be detected in blood samples from patients undergoing treatment. Given the challenges associated with diagnosing t-NEPC based on clinical criteria alone, identifying reliable biomarkers such as circPCDH11Y could greatly enhance early diagnosis and guide treatment decisions. Although this study provides novel insights into the role of circRNAs in prostate cancer transdifferentiation, particularly circPCDH11Y, several limitations remain. First, while our *in vitro* findings demonstrate the potential significance of circPCDH11Y in NED, validation in clinical samples is essential to establish its relevance in patients with t-NEPC. Second, the mechanistic details of how circPCDH11Y regulates neuroendocrine marker expression and AR signaling require further elucidation. Lastly, while we showed the role of circPCDH11Y in LNCaP cells, the lack of circPCDH11Y expression in DU145 cells may indicate that this circRNA, as the linear form of PCDH11Y, may be able to distinguish between AR-positive vs AR-negative neuroendocrine prostate cancers (57).

In conclusion, this preliminary study provides a foundation for understanding the role of circRNAs in prostate cancer progression, particularly in the context of neuroendocrine transdifferentiation. Our identification of circPCDH11Y as a novel, upregulated circRNA during NED opens the door for future studies aimed at validating its potential as a biomarker for t-NEPC. This could ultimately improve early diagnosis and offer new therapeutic strategies for managing treatment-resistant prostate cancer.

## Data Availability

Publicly available datasets were analyzed in this study. This data can be found here: NCBI GEO database, accession number GSE114052.

## Supplementary material

Supplementary Figure 1 Graphical representation of circPCDH11Y backsplicing site, qPCR primer annealing site, target amplification and Sanger Sequencing validation.

Supplementary Figure 2 Uncropped Western Blot analysis of positive and negative EVs markers TSG101 and Calnexin respectively at different stages of LNCaP neuroendocrine trans-differentiation.

Supplementary Figure 3 Graphical representation of circPCDH11Y selective amplification and silencing strategy. Both circPCDH11Y reverse primer and targeting DsiRNAs were synthetized in order to target the sequence encompassing the backsplicing junction site, so to elicit selective effects.

Supplementary Figure 4 Graphical representation of cell viability assay (right panels) and cell cycle distribution assay (left panels) performed on LNCaP cells following circPCDH11Y silencing.
