# Supplementary figures and images for: Co-modulation of a circular form of PCDH11Y during neuroendocrine differentiation of prostate cancer
Source: Front Oncol. 2025 Feb 11;15:1502405. doi: 10.3389/fonc.2025.1502405 (PMC11850525; doi:10.3389/fonc.2025.1502405)

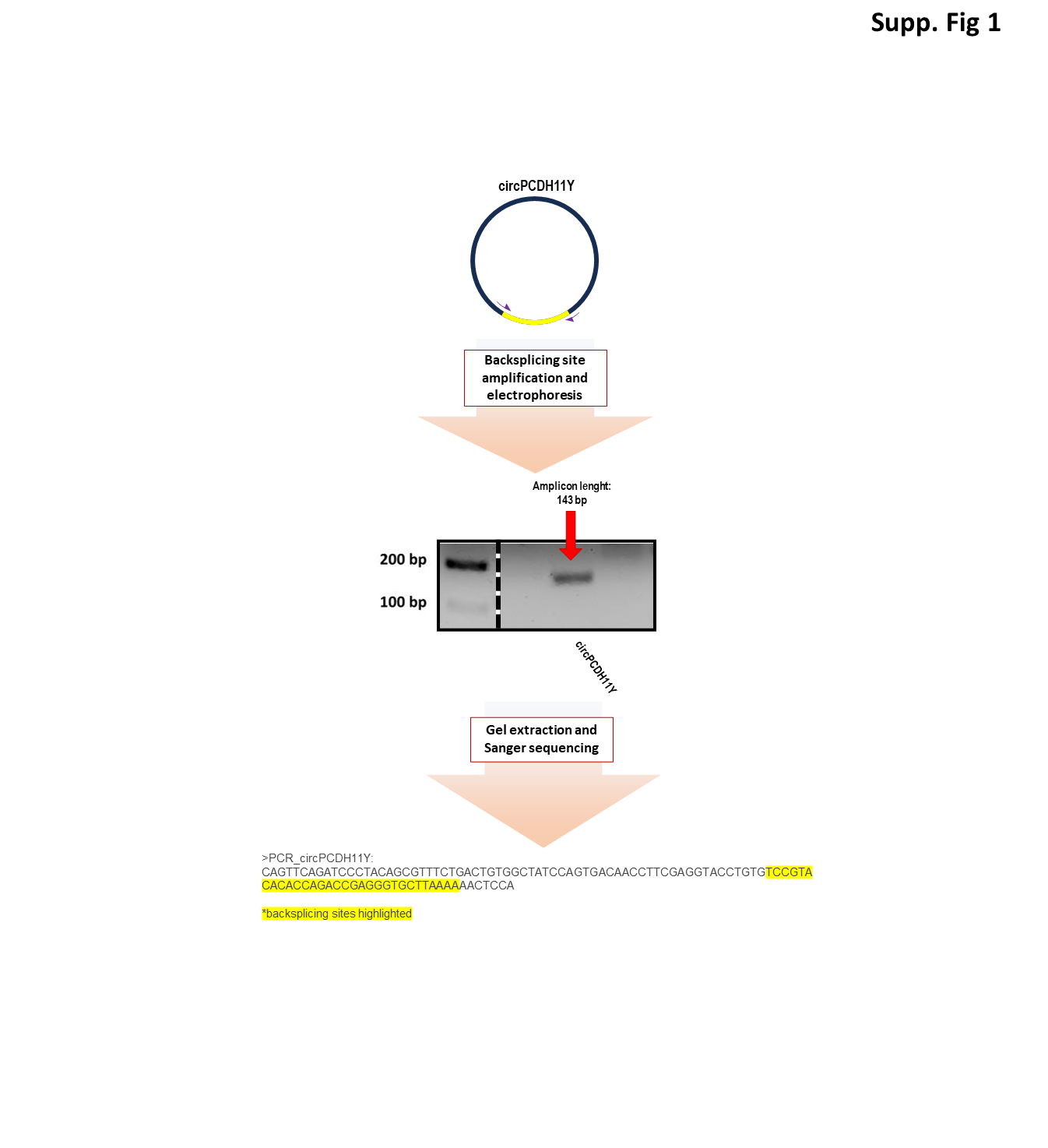

Supplement: Supplementary Figure 1 — Graphical representation of circPCDH11Y backsplicing site, qPCR primer annealing site, target amplification and Sanger Sequencing validation. [file Image1.tif]

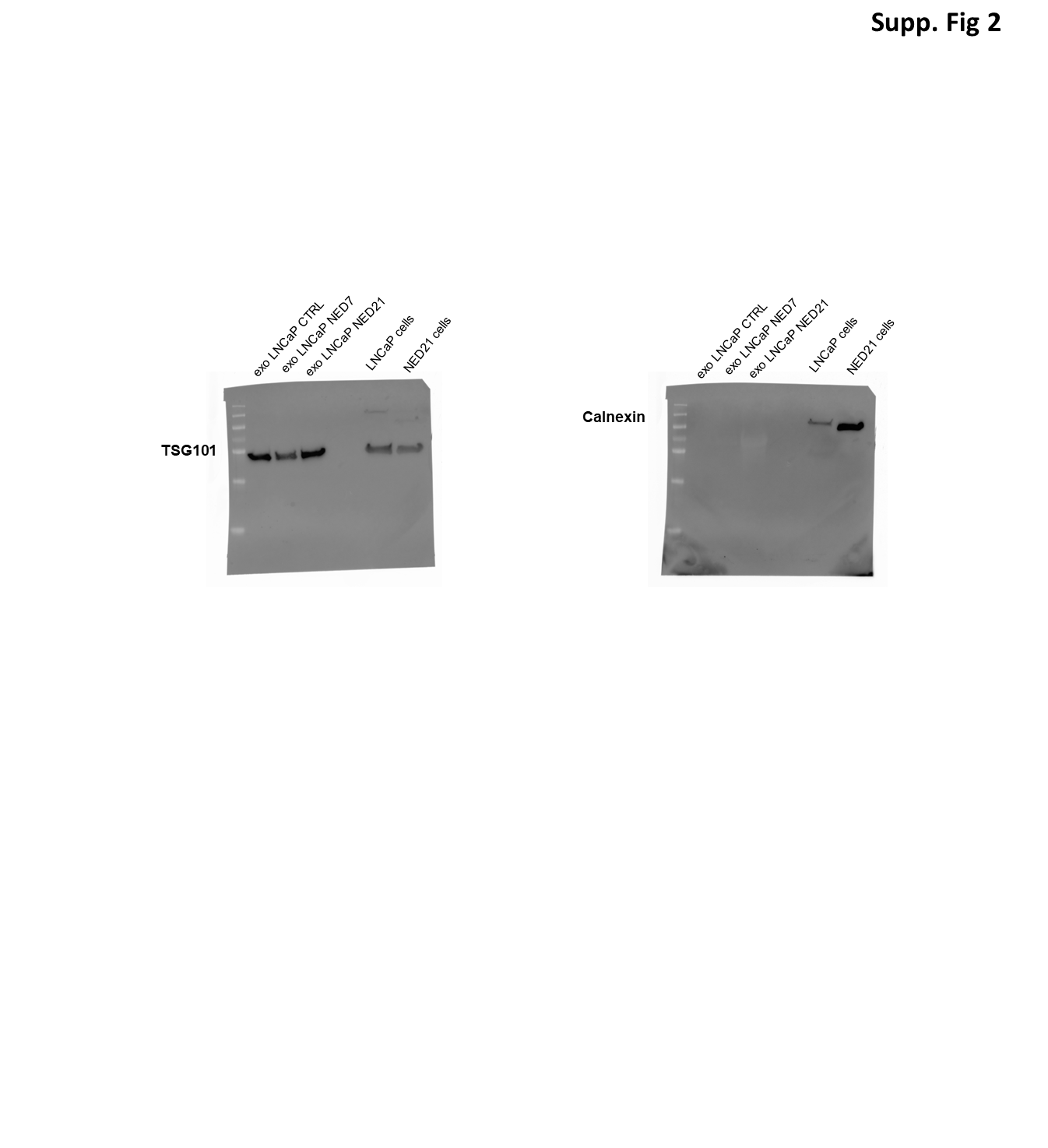

Supplement: Supplementary Figure 2 — Uncropped Western Blot analysis of positive and negative EVs markers TSG101 and Calnexin respectively at different stages of LNCaP neuroendocrine trans-differentiation. [file Image2.tif]

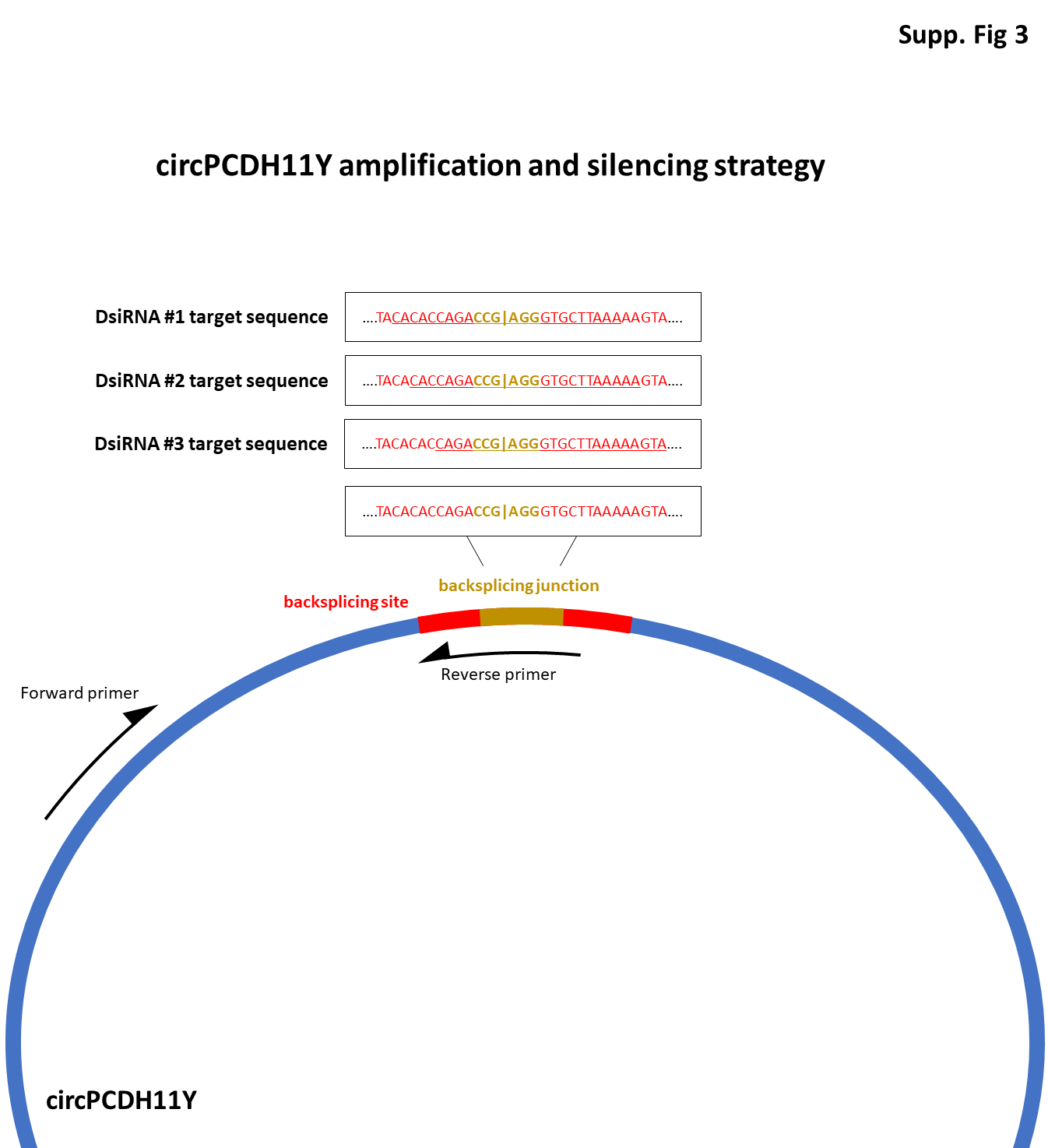

Supplement: Supplementary Figure 3 — Graphical representation of circPCDH11Y selective amplification and silencing strategy. Both circPCDH11Y reverse primer and targeting DsiRNAs were synthetized in order to target the sequence encompassing the backsplicing junction site, so to elicit selective effects. [file Image3.tif]

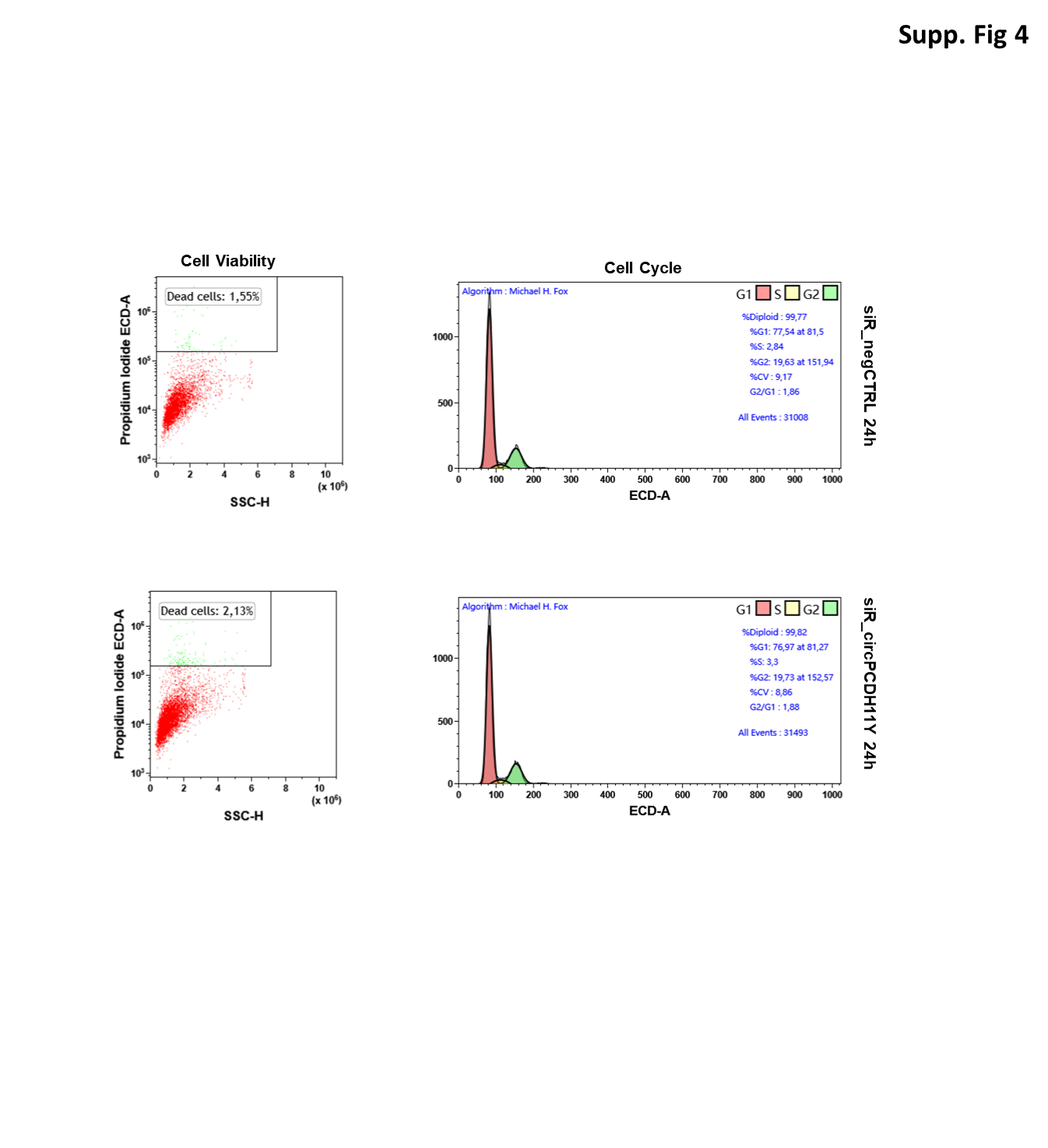

Supplement: Supplementary Figure 4 — Graphical representation of cell viability assay (right panels) and cell cycle distribution assay (left panels) performed on LNCaP cells following circPCDH11Y silencing. [file Image4.tif]
